# Data-driven subtyping of Alzheimer’s emergency presentations using unsupervised machine learning

**DOI:** 10.1016/j.ibneur.2026.08.016

**Published:** 2026-08-21

**Authors:** Tursun Alkam, Ebrahim Tarshizi, Andrew H. Van Benschoten

**Affiliations:** Master’s Program of Applied Artificial Intelligence, University of San Diego, San Diego, CA, USA

**Keywords:** Emergency department, Diagnostic clustering, Comorbidity profiling, Health informatics, Geriatric syndromes

## Abstract

**Background:**

Older adults with Alzheimer’s disease (AD) frequently present to emergency departments (EDs) with multiple coexisting conditions. However, age-specific patterns of multimorbidity in AD-related ED encounters remain incompletely characterized.

**Objective:**

To identify and describe age-specific multimorbidity subtypes among AD-associated ED encounters using unsupervised machine learning and heatmap-based diagnostic profiling.

**Methods:**

We conducted a cross-sectional analysis of the 2022 Nationwide Emergency Department Sample. AD-associated encounters were identified by an ICD-10-CM G30.x diagnosis code in any diagnosis field. The cohort included 125,461 ED encounters and was stratified into four age groups: 60–64, 65–74, 75–84, and ≥ 85 years. For each age group, the 30 most frequent co-occurring diagnoses were converted into binary indicators. KMeans clustering with eight clusters per age group was used for heatmap-based subtyping, while Uniform Manifold Approximation and Projection and Hierarchical Density-Based Spatial Clustering of Applications with Noise were used to visualize diagnostic structure.

**Results:**

Distinct multimorbidity profiles were identified across all age groups. Among adults aged 60–74 years, clusters commonly included psychiatric and metabolic conditions, such as depression, anxiety, diabetes, chronic kidney disease, substance-use diagnoses, and dehydration. Among adults aged ≥ 75 years, clusters more frequently included cardiorenal disease, urinary tract infection, metabolic encephalopathy, respiratory failure, do-not-resuscitate status, and palliative care. The cohort had a mean age of 81.7 ± 7.2 years, and 62.6% of encounters involved women.

**Conclusion:**

Patterns of coexisting conditions in AD-related ED visits differed substantially by age. Adults aged 60–74 years more often showed psychiatric and metabolic combinations, whereas those aged ≥ 75 years more often showed infection, cardiorenal disease, respiratory failure, and end-of-life care markers. Recognizing these age-related patterns may help clinicians anticipate common care needs when evaluating patients with AD in the ED.

## Highlights

• Identified age-specific multimorbidity subtypes in Alzheimer’s emergency visits.
• Applied interpretable unsupervised learning with heatmap-based cluster profiling.
• Revealed psychiatric–metabolic clusters in younger-old adults with AD.
• Frailty, infection, and end-of-life patterns dominated the oldest-old groups.
• Findings support precision triage and age-tailored emergency care strategies.

## 1 Introduction

Alzheimer’s disease (AD) contributes substantially to emergency department (ED) utilization among older adults, particularly during advanced stages characterized by cognitive decline, multimorbidity, and increased reliance on acute care systems (Gerlach et al., 2023, Anon, 2024, LaMantia et al., 2016, Phelan et al., 2012). These ED visits often reflect a convergence of chronic disease burden, acute decompensation, and social or caregiving insufficiencies, posing considerable challenges for triage, care planning, and outcome prediction (Gettel et al., 2024, Valeriani, 2011, Goldberg and Chavin, 1997).

While prior research has identified frequently co-occurring diagnoses among AD patients, such as hypertension, diabetes, urinary tract infection (UTI), and depression (Bynum et al., 2004, Rudolph et al., 2010, Camacho-Soto et al., 2025, Brewer et al., 2025, Alkam et al., 2025), most studies have examined these comorbidities in isolation or through predefined indices (Charlson et al., 1987, Stirland et al., 2025, Hassaine et al., 2020, Yourman et al., 2020, Snowden et al., 2017). Such approaches may obscure the complex interplay and clustering of diagnoses that define real-world emergency presentations. Additionally, they rarely capture how these patterns evolve across the aging continuum, from early cognitive decline to terminal frailty (Morandi et al., 2013, Pal and Manning, 2014, Pinardi et al., 2024). A more granular, data-driven understanding of diagnostic clustering in Alzheimer’s ED visits is needed to advance person-centered and age-tailored care strategies (Kojima et al., 2018, Bernini et al., 2025, Kumar et al., 2024).

To address this gap, we applied an unsupervised machine learning approach to the 2022 Nationwide Emergency Department Sample (NEDS) to identify clinically meaningful multimorbidity subtypes in AD-related ED presentations. We focused on four late-life age groups (60–64, 65–74, 75–84, and ≥85 years), constructing binary comorbidity matrices based on each group’s 30 most frequent ICD−10 diagnosis codes. These matrices were used to calculate within-cluster diagnostic loadings, with heatmaps visualizing conditions that were highly prevalent (≥40%) or defining (100%) within each cluster. This enabled a transparent, empirically grounded framework for thematic interpretation.

To support the construct validity of the heatmap-based clusters, we applied Uniform Manifold Approximation and Projection (UMAP) for dimensionality reduction and Hierarchical Density-Based Spatial Clustering of Applications with Noise (HDBSCAN) to explore latent structure in high-dimensional diagnostic space (Campello and Moulavi, 2013, Blanco-Portals et al., 2022, Yang et al., 2021). This secondary clustering was not used for label generation but provided a confirmatory lens into the distribution and heterogeneity of multimorbidity profiles.

Our thematic analysis of clusters, based on anchor diagnoses and high-loading comorbidities, revealed distinct, age-stratified patterns. Younger-old adults often presented with psychiatric-metabolic clusters involving depression, anxiety, substance use, and early renal disease, while the oldest-old were dominated by frailty phenotypes characterized by infection, cardiopulmonary compromise, palliative care, and do-not-resuscitate (DNR) status. These patterns suggest a clinically coherent typology of AD-related ED presentations that evolves across the lifespan (Covinsky et al., 2003, Fick et al., 2002, Elahi and Miller, 2017).

By integrating heatmap analytics with unsupervised machine learning, this study offers a scalable, interpretable framework for age-sensitive subtyping in AD emergency care. Such phenotypic stratification may ultimately support precision triage, resource targeting, and clinical decision-making in acute settings where time, context, and cognitive impairment complicate care delivery (Topaz et al., 2019, Strehlow et al., 2024, Raff et al., 2024, Mohammadi et al., 2023).

## 2 Methods

### 2.1 Data source and study sample

We conducted a cross-sectional analysis using the 2022 Healthcare Cost and Utilization Project Nationwide Emergency Department Sample (NEDS), a nationally representative database of hospital-based ED visits in the United States. AD-associated encounters were identified by the presence of an ICD−10-CM G30.x diagnosis code in any available diagnosis field.

The dataset included 125,461 AD-associated ED encounters. Age was available for 125,453 encounters; eight encounters (<0.01%) had missing age and were therefore excluded from age-stratified analyses but retained in analyses that did not require age. Encounters with available age were stratified into four prespecified groups: 60–64 years (n = 2784), 65–74 years (n = 18,512), 75–84 years (n = 51,440), and ≥ 85 years (n = 52,717). These strata were selected to represent clinically interpretable stages across later life while maintaining sufficient sample sizes for age-specific analysis. This grouping allowed comparison of multimorbidity patterns from younger-old adults through the oldest-old, among whom the prevalence and combinations of chronic disease, frailty, and acute medical conditions may differ.

### 2.2 Dimensionality reduction and unsupervised clustering visualization

To capture the latent structure in the high-dimensional comorbidity data, we applied UMAP for nonlinear dimensionality reduction and a binary matrix created for clustering. The resulting 2-dimensional embeddings provided a compressed representation of diagnostic co-occurrence patterns for clustering and visualization. Then, HDBSCAN was applied to identify natural groupings of ED presentations within each age group. HDBSCAN is well-suited for variable-density clusters and does not require pre-specifying the number of clusters. HDBSCAN was implemented with a min_cluster_size of 50 to ensure that identified clusters represented clinically meaningful subgroups rather than small, noise-driven partitions. Because real-world ED presentations are heterogeneous, cluster sizes are expected to be imbalanced; we therefore report cluster prevalence and interpret small clusters cautiously. HDBSCAN was used primarily to explore and visualize latent structure in the UMAP space (rather than to define the final diagnosis-based subtypes).

#### 2.2.1 Top 30 ICD−10 code selection

For each age group, we identified the 30 most frequently occurring ICD−10 diagnostic codes within the AD cohort. These were selected to ensure focus on clinically relevant and highly prevalent comorbidities in each stratum, and served as the dimensional features for subsequent clustering analysis. Diagnoses were included regardless of their order in the diagnosis field (i10_dx1 to i10_dx40). The top 30 most frequent ICD−10-CM diagnosis codes within each age group were selected to balance comprehensiveness with interpretability. This threshold ensured that high-prevalence conditions shaping ED presentations were included while minimizing noise from rare diagnoses that might hinder visualization and thematic labeling. We acknowledge that less common but clinically important conditions could be excluded, a limitation addressed in the Discussion. This threshold balances clinical interpretability (heatmap readability) with adequate coverage of common comorbidity burden while limiting sparsity from low-frequency diagnoses that can destabilize clustering. Sensitivity checks using alternative cutoffs (e.g., top 20 and top 40) yielded similar thematic cluster profiles, supporting the robustness of the top−30 specification. We did not apply PCA prior to primary diagnosis-defined clustering to preserve direct clinical interpretability; dimensionality reduction was used mainly for visualization.

### 2.3 Heatmap-based comorbidity cluster labeling and interpretation

A binary matrix was constructed for each age group, where rows represented individual ED visits and columns represented the top 30 ICD−10 codes. Each cell was coded as 1 if the corresponding diagnosis was present and 0 otherwise. This matrix formed the input for dimensionality reduction and clustering analyses.

To determine the optimal number of clusters, we applied the elbow method by plotting the within-cluster sum of squares (inertia) against a range of k values (2−9) for each age group (Supplementary Figure 1). While no sharply defined inflection point emerged, a choice of *k = 8* was made for all age groups to ensure consistency across timepoints and to maintain interpretability. This decision balances model parsimony with sufficient complexity to capture clinically relevant multimorbidity patterns. Furthermore, choosing a uniform k facilitated comparative analyses of cluster evolution over time without introducing variability due to differing model structures. KMeans clustering (k = 8) served as the primary method for heatmap-based comorbidity subtyping and labeling; elbow and silhouette inspection were used to support this choice. Internal validation metrics across k = 2–9 are provided in Supplementary Table 1.

To characterize the composition of each cluster, we calculated diagnosis loadings, which represent the proportion of ED visits within a cluster that contained a specific ICD−10 diagnosis code. A loading value of 1.00 indicated that a particular diagnosis was present in 100% of the visits within that cluster, thereby identifying it as an index comorbidity, a potential hallmark of that cluster. A loading threshold of ≥ 0.40 was selected to identify diagnoses present in at least 40% of visits within a cluster. This cutoff was chosen based on exploratory inspection of the heatmaps to capture major contributing diagnoses without diluting the cluster profiles with marginal contributors. Testing alternative thresholds (0.30 and 0.50) produced similar thematic patterns, suggesting that the results are robust to modest variation in this parameter. Therefore, diagnoses with loading values ≥ 0.40 (i.e., present in at least 40% of the visits in the cluster) were retained for deeper interpretation, as these were deemed to meaningfully contribute to the clinical character of the cluster.

These loadings were visualized using annotated heatmaps, where each row represented a diagnostic code, each column represented a cluster, and cell colors reflected loading values (from 0 to 1.00). This approach allowed for rapid identification of dominant and co-occurring diagnoses in each cluster. For instance, in a cluster with loading = 1.00 for dehydration (E86.0) and high loadings (≥0.40) for urinary tract infection (N39.0), hypotension (I95.9), and DNR status (Z66), the cluster likely represents an older adult with AD presenting in acute medical decompensation, possibly related to infection, volume loss, and end-of-life status.

By applying these heatmap-driven interpretations across age groups, we were able to derive clinically meaningful multimorbidity profiles, even in the absence of pre-defined disease categories.

### 2.4 Clinical THematic Labeling

The highest-loading diagnoses for each cluster were independently reviewed, and descriptive clinical themes, such as ‘frailty with acute infection’ or ‘psychiatric-metabolic overlap’ or ‘end-stage cardio-renal disease’, were assigned based on these patterns. The goal was to derive age-stratified comorbidity subtypes of AD-related ED visits based on the unsupervised clusters.

### 2.5 Software and reproducibility

All analyses were conducted in Python 3.10 using Google Colab. Libraries included: pandas, numpy, umap-learn, hdbscan, matplotlib, and seaborn. Reproducibility notebooks are available at https://github.com/TAlkam/NEDS

## 3 Results

### 3.1 Study population and demographic and socioeconomic characteristics

The study included 125,461 AD-associated ED encounters. Age was available for 125,453 encounters; eight encounters (<0.01%) had missing age and could not be assigned to an age stratum. Among encounters with available age, 2784 (2.2%) involved adults aged 60–64 years, 18,512 (14.8%) involved adults aged 65–74 years, 51,440 (41.0%) involved adults aged 75–84 years, and 52,717 (42.0%) involved adults aged ≥ 85 years. The mean age of the overall cohort was 81.7 ± 7.2 years (Table 1).

**Table 1:** Demographic and socioeconomic characteristics of Alzheimer’s disease emergency department encounters by age group, NEDS 2022.

Characteristic	Overall AD cohort (N = 125,461)	60–64 (n = 2784)	65–74 (n = 18,512)	75–84 (n = 51,440)	≥ 85 (n = 52,717)
Age, years, mean ± SD	81.7 ± 7.2	62.2 ± 1.3	70.6 ± 2.7	79.9 ± 2.8	88.3 ± 1.8
Age missing	8 (<0.01)	—	—	—	—
Sex					
Male	46,973 (37.4)	1288 (46.3)	8032 (43.4)	20,350 (39.6)	17,298 (32.8)
Female	78,483 (62.6)	1496 (53.7)	10,479 (56.6)	31,089 (60.4)	35,416 (67.2)
Missing	5 (<0.01)	0	1 (<0.01)	1 (<0.01)	3 (<0.01)
Race/ethnicity					
White	88,567 (70.6)	1790 (64.3)	12,362 (66.8)	36,816 (71.6)	37,597 (71.3)
Black	14,966 (11.9)	493 (17.7)	3043 (16.4)	5866 (11.4)	5564 (10.6)
Hispanic	12,997 (10.4)	297 (10.7)	1818 (9.8)	5200 (10.1)	5682 (10.8)
Asian/Pacific Islander	3235 (2.6)	49 (1.8)	333 (1.8)	1193 (2.3)	1660 (3.1)
Native American	275 (0.2)	14 (0.5)	64 (0.3)	121 (0.2)	76 (0.1)
Other	3148 (2.5)	71 (2.6)	498 (2.7)	1315 (2.6)	1264 (2.4)
Missing	2273 (1.8)	70 (2.5)	394 (2.1)	929 (1.8)	874 (1.7)
Primary payer					
Medicare	111,130 (88.6)	1462 (52.5)	15,835 (85.5)	45,824 (89.1)	48,002 (91.1)
Medicaid	2480 (2.0)	596 (21.4)	416 (2.2)	813 (1.6)	655 (1.2)
Private insurance	8148 (6.5)	547 (19.6)	1571 (8.5)	3268 (6.4)	2761 (5.2)
Self-pay	999 (0.8)	86 (3.1)	185 (1.0)	410 (0.8)	318 (0.6)
No charge	23 (<0.1)	5 (0.2)	5 (<0.1)	9 (<0.1)	4 (<0.1)
Other	2637 (2.1)	85 (3.1)	492 (2.7)	1097 (2.1)	963 (1.8)
Missing	44 (<0.1)	3 (0.1)	8 (<0.1)	19 (<0.1)	14 (<0.1)
ZIP-code median household income quartile					
Quartile 1 (lowest)	32,608 (26.0)	896 (32.2)	5571 (30.1)	13,626 (26.5)	12,515 (23.7)
Quartile 2	31,627 (25.2)	765 (27.5)	4871 (26.3)	13,154 (25.6)	12,831 (24.3)
Quartile 3	30,619 (24.4)	627 (22.5)	4276 (23.1)	12,445 (24.2)	13,270 (25.2)
Quartile 4 (highest)	29,276 (23.3)	468 (16.8)	3537 (19.1)	11,682 (22.7)	13,589 (25.8)
Missing	1331 (1.1)	28 (1.0)	257 (1.4)	533 (1.0)	512 (1.0)
Patient residence					
Large central metropolitan	34,934 (27.8)	747 (26.8)	4956 (26.8)	13,711 (26.7)	15,520 (29.4)
Large fringe metropolitan	29,028 (23.1)	621 (22.3)	4122 (22.3)	12,091 (23.5)	12,194 (23.1)
Medium metropolitan	28,468 (22.7)	646 (23.2)	4223 (22.8)	11,713 (22.8)	11,886 (22.5)
Small metropolitan	13,841 (11.0)	307 (11.0)	2068 (11.2)	5850 (11.4)	5614 (10.6)
Micropolitan	11,253 (9.0)	253 (9.1)	1817 (9.8)	4633 (9.0)	4549 (8.6)
Noncore	7759 (6.2)	199 (7.1)	1279 (6.9)	3375 (6.6)	2901 (5.5)
Missing	178 (0.1)	11 (0.4)	47 (0.3)	67 (0.1)	53 (0.1)
Housing status					
ICD−10-CM–documented homelessness	174 (0.1)	26 (0.9)	77 (0.4)	52 (0.1)	19 (<0.1)

**Note:** Values are presented as n (%) unless otherwise indicated. Percentages were calculated using the total number of encounters in each column as the denominator. Eight encounters had missing age and were included in the overall cohort but could not be assigned to an age stratum. Race/ethnicity represents the HCUP combined race/ethnicity variable. ZIP-code median household income quartile reflects the estimated median household income of the patient’s residential ZIP code. Homelessness was identified from ICD−10-CM Z59.0x diagnosis codes and therefore represents documented homelessness rather than a directly collected housing-status measure. Percentages may not total 100% because of rounding. AD, Alzheimer’s disease; ED, emergency department; HCUP, Healthcare Cost and Utilization Project; NEDS, Nationwide Emergency Department Sample; SD, standard deviation.

Women accounted for 62.6% of encounters overall, increasing from 53.7% among adults aged 60–64 years to 67.2% among those aged ≥ 85 years. Overall, 70.6% of encounters were recorded as White, 11.9% as Black, 10.4% as Hispanic, 2.6% as Asian/Pacific Islander, 0.2% as Native American, and 2.5% as another race or ethnicity; race/ethnicity was missing for 1.8% of encounters. The proportion of encounters recorded as Black decreased from 17.7% in the 60–64-year group to 10.6% in the ≥ 85-year group (Table 1).

Medicare was the primary expected payer for 88.6% of encounters overall, increasing from 52.5% among adults aged 60–64 years to 91.1% among those aged ≥ 85 years. Among adults aged 60–64 years, Medicaid and private insurance accounted for 21.4% and 19.6% of encounters, respectively, compared with 1.2% and 5.2%, respectively, among adults aged ≥ 85 years (Table 1).

The proportion of encounters from ZIP codes in the lowest median household income quartile decreased from 32.2% among adults aged 60–64 years to 23.7% among those aged ≥ 85 years. Conversely, the proportion from ZIP codes in the highest income quartile increased from 16.8% to 25.8% across these age groups. Approximately half of the encounters in each age group involved residents of large central or large fringe metropolitan areas. ICD−10-CM-documented homelessness was present in 174 encounters (0.1%) overall, including 26 encounters (0.9%) among adults aged 60–64 years, 77 (0.4%) among those aged 65–74 years, 52 (0.1%) among those aged 75–84 years, and 19 (<0.1%) among those aged ≥ 85 years (Table 1).

### 3.2 UMAP-HDBSCAN visualization and internal cluster validation

**Fig. 1:**
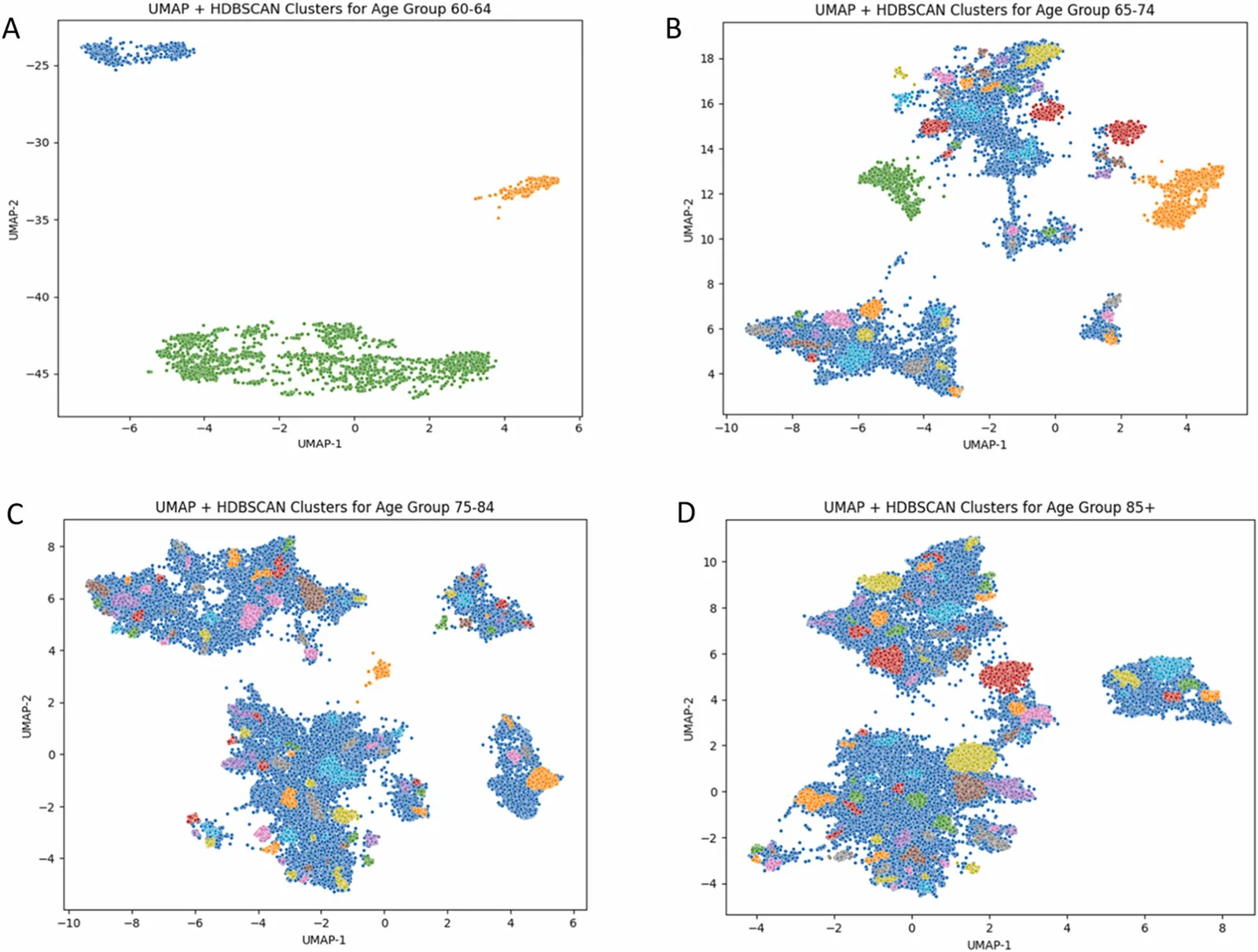
UMAP + HDBSCAN visualization of comorbidity clusters. UMAP dimensionality reduction with HDBSCAN clustering reveals diagnostic code clusters among AD in ED visits, stratified by age group: **(A)** 60–64, **(B)** 65–74, **(C)** 75–84, and **(D)** 85 +. Each point represents a visit, color-coded by diagnostic similarity based on ICD−10 co-occurrence. Younger groups (A–B) show more distinct and well-separated clusters, while older groups (C–D) display greater overlap and fragmentation, reflecting increasing multimorbidity complexity and diagnostic convergence with age. These visualizations support age-related shifts in comorbidity architecture among ED patients with AD.

UMAP-HDBSCAN visualization demonstrated multiple regions of diagnostic co-occurrence within each age stratum (Fig. 1). The 60–64-year embedding contained three broadly separated regions, whereas the embeddings for the older age groups contained a larger number of distributed and overlapping regions. HDBSCAN cluster assignments were used for visualization and were distinct from the eight-cluster KMeans solutions used for the heatmap profiles.

Across k values ranging from 2 to 9, within-cluster inertia decreased progressively in all four age groups without a sharply defined inflection point (Supplementary Figure 1). At k = 8, silhouette coefficients were 0.0921 for ages 60–64 years, 0.0659 for ages 65–74 years, 0.0652 for ages 75–84 years, and 0.0764 for ages ≥ 85 years. The corresponding Calinski-Harabasz values were 115.65, 740.05, 2269.34, and 2262.78, respectively, and the Davies-Bouldin indices were 2.7474, 2.9563, 2.8986, and 2.7202, respectively (Supplementary Table 1). The age-specific frequencies of the 30 ICD−10-CM codes included in the heatmap analyses are presented in Supplementary Tables 2–5.

### 3.3 Heatmap cluster profiles among adults aged 60–64 years

**Fig. 2:**
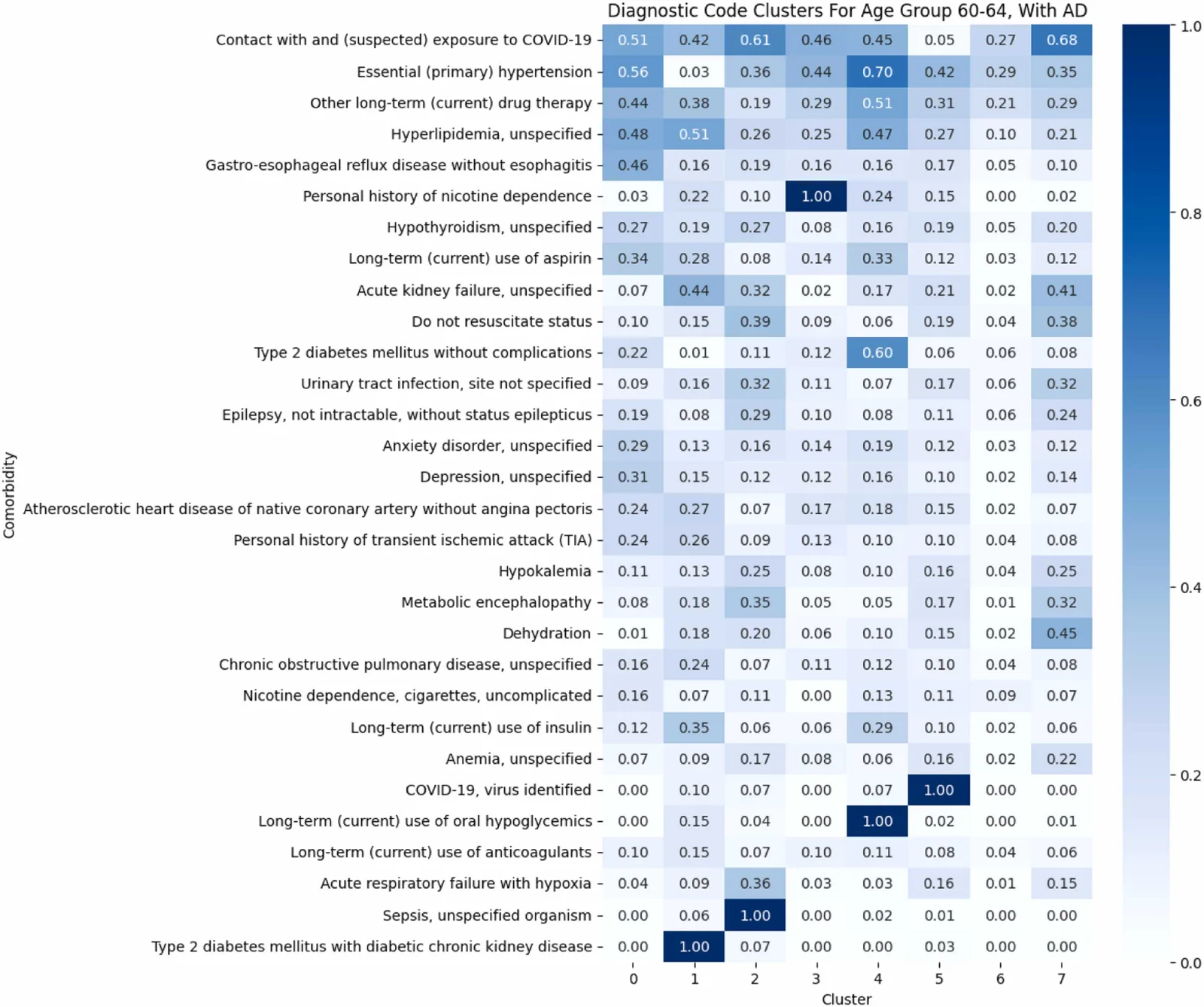
Heatmap of diagnostic clusters among adults aged 60–64 with AD in the ED. Each column represents a cluster derived from the top 30 most frequent ICD−10 diagnoses for AD-related ED visits in this age group (NEDS 2022). Each cell displays the proportion of visits within the cluster that contained the corresponding diagnosis (loading). Darker blue indicates higher prevalence, with a value of 1.00 signifying a defining comorbidity for that cluster. Notable themes include psychiatric–metabolic overlap, substance use, and early renal and cardiovascular disease.

Eight heatmap clusters were generated for the 60–64-year group. Seven clusters showed identifiable diagnostic profiles, whereas Cluster 7 had no comparably strong diagnostic signal (Fig. 2; Table 2).

**Table 2:** Cluster summary from heatmap analysis.

Age Group	Cluster	Top Comorbidities
60–64	0	Hypertension, COVID−19 contact, Hyperlipidemia, Drug therapy, Nicotine dependence
	1	Metabolic encephalopathy, GERD, Hypertension, Hyperlipidemia
	2	Depression, Anxiety, Substance use, Hypokalemia
	3	T2DM with CKD, Hypertension Stage 1–4, Drug therapy
	4	Dehydration, GERD, COVID−19 contact, Hyperlipidemia
	5	Encounter for palliative care, Anemia, CKD stage 3, Diabetes with CKD
	6	UTI, Atrial fibrillation, Heart failure, COPD
	7	None (weak cluster signal)
65–74	0	Hyperlipidemia, Drug therapy, Hypertension
	1	Depression, Nicotine dependence, COVID−19 contact
	2	Dehydration, Hyponatremia, Anticoagulants, GERD
	3	T2DM with CKD, CKD stage 3, Hypertension Stage 1–4
	4	Hypokalemia, Encephalopathy, Diabetes with CKD
	5	Palliative care, Anemia, Respiratory failure
	6	Atrial fibrillation, Heart failure, COPD
	7	COVID−19 contact, Depression
75–84	0	Do Not Resuscitate, Metabolic encephalopathy, COVID−19 contact
	1	Hypertension, Hyperlipidemia, Drug therapy, GERD
	2	Anxiety, Depression, Nicotine use, Hypokalemia
	3	Heart failure, GERD, COVID−19, Encephalopathy
	4	Diabetes with CKD, CKD Stage 3, Hypokalemia
	5	Atrial fibrillation, Anemia, COPD, TIA
	6	Palliative care, UTI, Respiratory failure
	7	GERD, CKD, Depression
85 +	0	Hypokalemia, COVID−19, DNR, Encephalopathy
	1	Hypertension with HF, Diabetes, Hyperlipidemia
	2	Atrial fibrillation, GERD, Drug therapy
	3	Diabetes with CKD, CKD stage 3, Anticoagulants
	4	COPD, Encephalopathy, HTN Stage 1–4, Hypokalemia
	5	UTI, Palliative care, Respiratory failure
	6	Nicotine dependence, Anxiety, Hypothyroidism
	7	COVID−19 contact, Depression, Hypertension

Cluster 0 included hypertension, contact with or suspected exposure to COVID−19, hyperlipidemia, long-term drug therapy, and nicotine dependence. Cluster 1 included metabolic encephalopathy, gastroesophageal reflux disease (GERD), hypertension, and hyperlipidemia. Cluster 2 included depression, anxiety, substance-use diagnoses, and hypokalemia. Cluster 3 included type 2 diabetes mellitus with chronic kidney disease (CKD), hypertensive CKD, and long-term drug therapy.

Cluster 4 included dehydration, GERD, contact with or suspected exposure to COVID−19, and hyperlipidemia. Cluster 5 included an encounter for palliative care, anemia, stage 3 CKD, and diabetes with CKD. Cluster 6 included urinary tract infection (UTI), atrial fibrillation, heart failure, and chronic obstructive pulmonary disease (COPD) (Fig. 2; Table 2).

### 3.4 Heatmap cluster profiles among adults aged 65–74 years

**Fig. 3:**
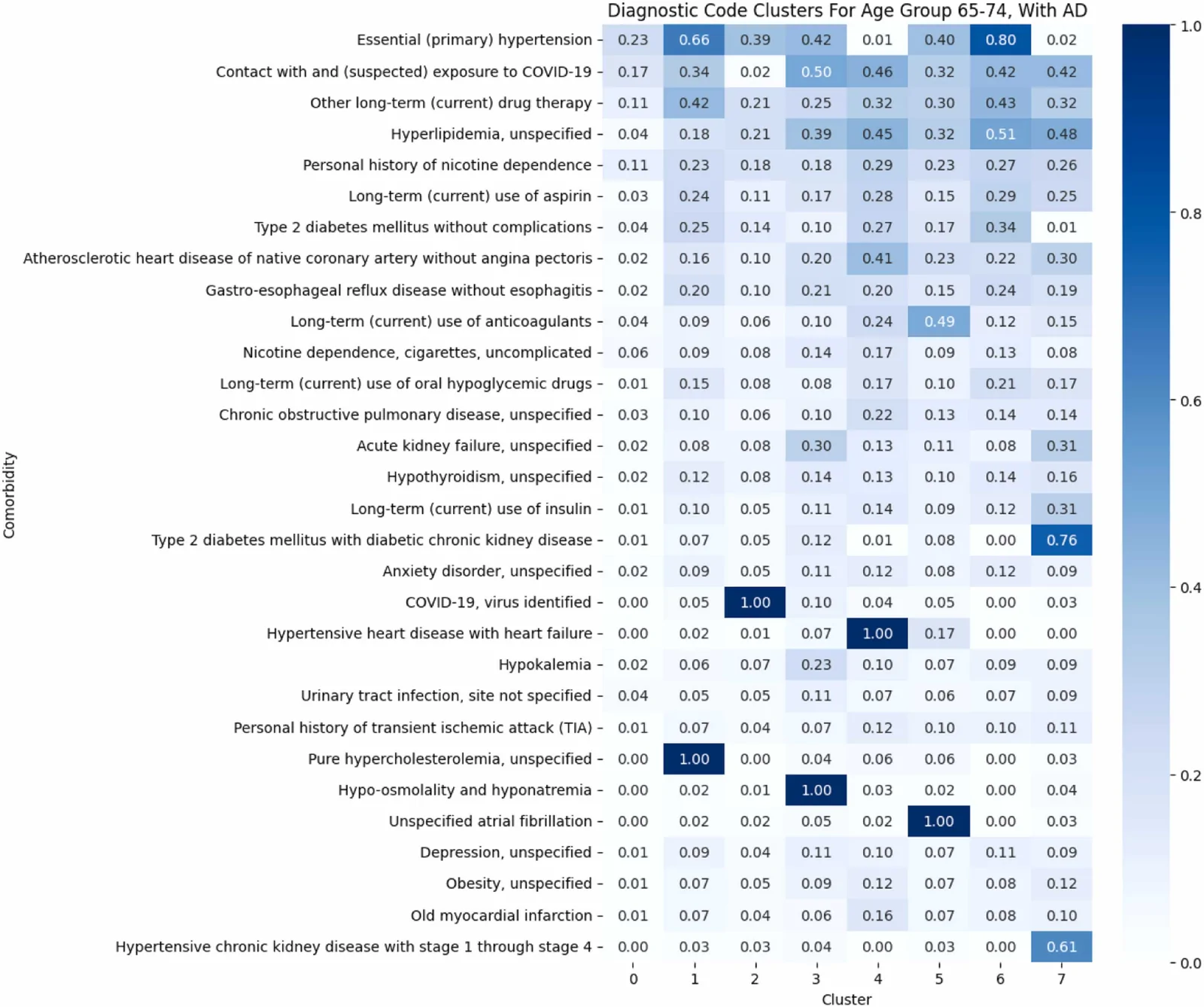
Heatmap of diagnostic clusters among adults aged 65–74 with AD in the ED. This heatmap displays the distribution of the 30 most frequent ICD−10 diagnoses across clusters derived for AD-related ED visits in the 65–74 age group (NEDS 2022). Each cell indicates the diagnosis loading (proportion of visits within a cluster with the comorbidity), with a value of 1.00 indicating a defining condition. Notable patterns include clusters anchored by COVID−19, metabolic syndrome, diabetic kidney disease, hypokalemia, and atrial fibrillation—suggesting a shift toward cardio-metabolic frailty and chronic disease burden with acute destabilization.

Eight diagnostic profiles were observed among adults aged 65–74 years (Fig. 3; Table 2). Cluster 0 included hyperlipidemia, long-term drug therapy, and hypertension. Cluster 1 included depression, nicotine dependence, and contact with or suspected exposure to COVID−19. Cluster 2 included dehydration, hyponatremia, anticoagulant use, and GERD. Cluster 3 included diabetes with CKD, stage 3 CKD, and hypertensive CKD.

Cluster 4 included hypokalemia, metabolic encephalopathy, and diabetes with CKD. Cluster 5 included palliative care, anemia, and respiratory failure. Cluster 6 included atrial fibrillation, heart failure, and COPD. Cluster 7 included contact with or suspected exposure to COVID−19 and depression (Fig. 3; Table 2).

### 3.5 Heatmap cluster profiles among adults aged 75–84 years

**Fig. 4:**
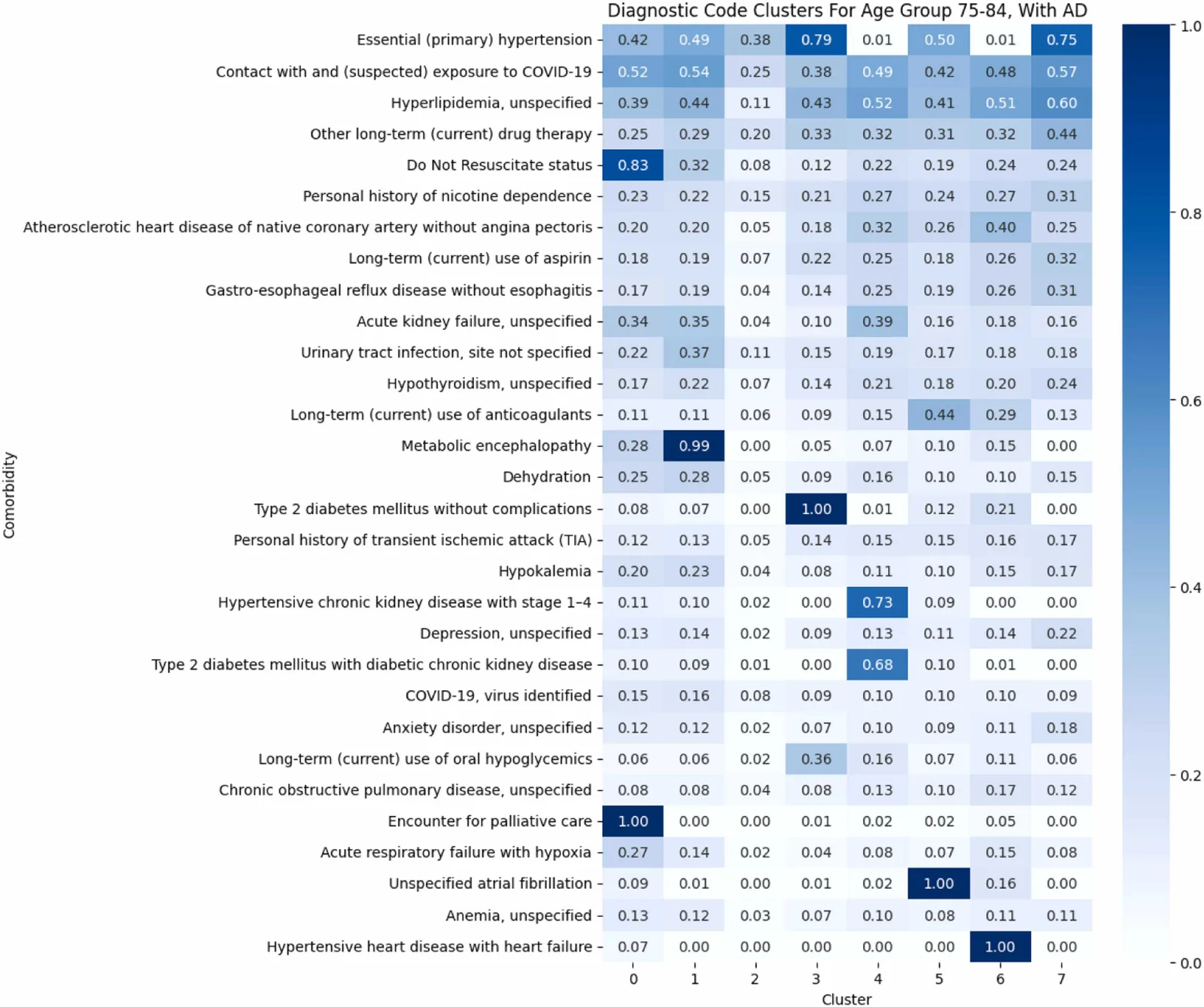
Heatmap of diagnostic clusters among adults aged 75–84 with AD in the ED. This heatmap presents the loading values of the top 30 ICD−10 comorbidities across diagnostic clusters derived for AD-related ED visits in the 75–84 age group (NEDS 2022). Several clusters are anchored by high-prevalence conditions such as metabolic encephalopathy, type 2 diabetes, chronic kidney disease, palliative care encounters, and DNR status. The patterns suggest convergence of chronic cardiovascular, renal, and neurologic conditions with markers of acute decompensation and terminal decline.

Eight clusters containing different combinations of chronic and acute diagnoses were observed among adults aged 75–84 years (Fig. 4; Table 2). Cluster 0 included do-not-resuscitate (DNR) status, metabolic encephalopathy, and contact with or suspected exposure to COVID−19. Cluster 1 included hypertension, hyperlipidemia, long-term drug therapy, and GERD. Cluster 2 included anxiety, depression, nicotine use, and hypokalemia. Cluster 3 included heart failure, GERD, COVID−19, and metabolic encephalopathy.

Cluster 4 included diabetes with CKD, stage 3 CKD, and hypokalemia. Cluster 5 included atrial fibrillation, anemia, COPD, and a history of transient ischemic attack. Cluster 6 included palliative care, UTI, and respiratory failure. Cluster 7 included GERD, CKD, and depression (Fig. 4; Table 2).

### 3.6 Heatmap cluster profiles among adults aged ≥ 85 years

**Fig. 5:**
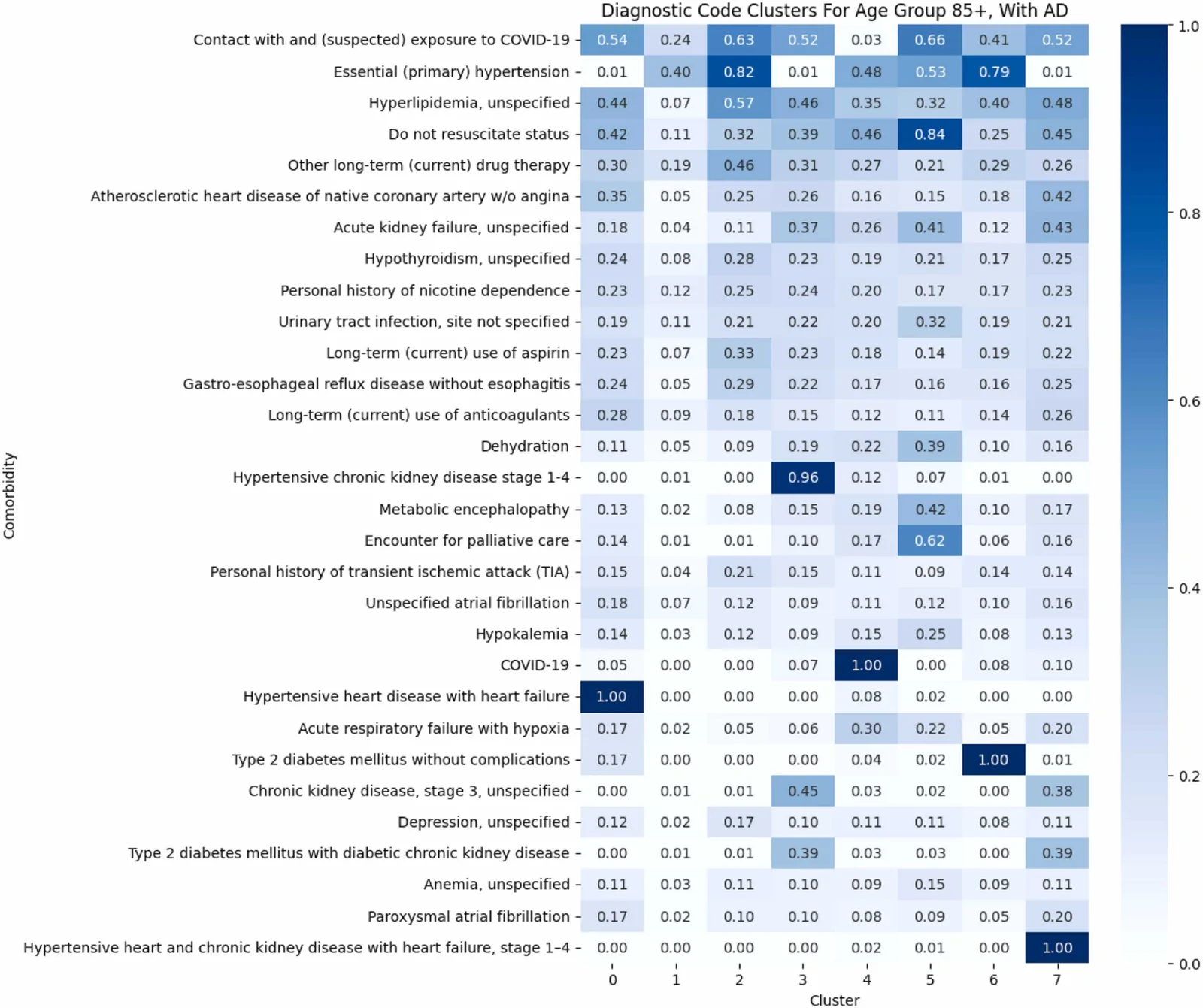
Heatmap of diagnostic clusters among adults aged 85 and older with AD in the ED. This heatmap displays diagnosis loadings for the top 30 comorbidities across eight data-driven clusters for patients aged 85 + with AD-related ED visits (NEDS 2022). Clusters reflect terminal multimorbidity patterns with high representation of conditions such as hypertensive heart failure, metabolic encephalopathy, chronic kidney disease, COVID−19, and do-not-resuscitate (DNR) status. The clustering highlights syndromes of end-of-life complexity and frailty, suggesting a transition from acute care toward palliative trajectories in this oldest-old population.

Eight heatmap profiles were identified among adults aged ≥ 85 years (Fig. 5; Table 2). Cluster 0 included hypokalemia, COVID−19, DNR status, and metabolic encephalopathy. Cluster 1 included hypertensive heart disease with heart failure, diabetes, and hyperlipidemia. Cluster 2 included atrial fibrillation, GERD, and long-term drug therapy. Cluster 3 included diabetes with CKD, stage 3 CKD, and anticoagulant use.

Cluster 4 included COPD, metabolic encephalopathy, hypertensive CKD, and hypokalemia. Cluster 5 included UTI, palliative care, and respiratory failure. Cluster 6 included nicotine dependence, anxiety, and hypothyroidism. Cluster 7 included contact with or suspected exposure to COVID−19, depression, and hypertension (Fig. 5; Table 2).

## 4 Discussion

### 4.1 Interpretation of age-specific multimorbidity patterns

This study identified age-related differences in the organization of multimorbidity among AD-associated ED encounters. Across the four age strata, the profiles shifted from psychiatric-metabolic and early cardiorenal combinations in younger adults toward infection-related decompensation, advanced cardiorenal and respiratory disease, and goals-of-care indicators in older adults. These findings reinforce that AD-related ED presentations are rarely defined by a single condition; instead, chronic disease, acute illness, and care-planning factors often occur together. The combined use of unsupervised clustering and heatmap-based diagnosis profiling provided an interpretable way to summarize this heterogeneity, with the age-specific diagnostic patterns and corresponding clinical themes presented in Table 2, Table 3.

**Table 3:** Main clinical themes derived from heatmap-based cluster profiles.

Age Group	Dominant Clinical Themes
60–64	- Early cardiometabolic burden (hypertension, hyperlipidemia) - Psychiatric comorbidities (depression, anxiety) - Substance use and nicotine dependence - Initial renal compromise (T2DM, CKD) - Acute decompensation (encephalopathy, GERD) in younger AD patients
65–74	- Increasing metabolic-renal overlap (T2DM with CKD, CKD Stage 3) - Neuropsychiatric clustering (depression, anxiety, nicotine dependence) - Acute-on-chronic instability (dehydration, hypokalemia, hyponatremia) - Emergence of frailty and palliative care markers
75–84	- Syndromic convergence: cognitive decompensation, cardiorenal dysfunction - Electrolyte imbalance (hypokalemia), infection vulnerability (COVID−19, UTI) - Respiratory failure and encephalopathy - Palliative trajectories and polypharmacy burden
85 +	- Terminal frailty phenotype with advanced multimorbidity (HF, CKD, COPD) - End-of-life clustering (Do Not Resuscitate, palliative care) - High prevalence of infection-related presentations (COVID−19, UTI) - Severe electrolyte and organ dysfunction (hypokalemia, encephalopathy)

Among adults aged 60–64 and 65–74 years, the recurrent co-occurrence of depression, anxiety, nicotine- or substance-related diagnoses, diabetes, chronic kidney disease (CKD), and electrolyte or volume disturbances suggests that neuropsychiatric and metabolic needs may converge early in the acute-care course of AD. Prior studies have similarly shown that dementia-related hospital and ED use is shaped by both chronic illness and neuropsychiatric burden (Bynum et al., 2004, Rudolph et al., 2010, Snowden et al., 2017). These profiles may identify encounters in which behavioral health assessment, medication review, and chronic disease management warrant simultaneous attention. However, because NEDS contains encounter-level administrative data, the present analysis cannot determine whether these conditions precipitated the ED visit or represented background comorbidity.

In the 75–84 and ≥ 85-year groups, the increasing concentration of CKD, heart failure, atrial fibrillation, urinary tract infection, metabolic encephalopathy, respiratory failure, do-not-resuscitate status, and palliative care is consistent with the reduced physiologic reserve and greater frailty that accompany advanced age and dementia (Pal and Manning, 2014, Kojima et al., 2018, Covinsky et al., 2003, Fick et al., 2002, Nobili et al., 2011, Antoniello et al., 2022, Kostakopoulos et al., 2021). The co-occurrence of infection, encephalopathy, electrolyte disturbance, and cardiopulmonary disease may reflect the multiple simultaneous care needs that complicate acute evaluation in older adults with AD. Do-not-resuscitate and palliative-care codes should not be interpreted as indicating that every encounter represented terminal care; rather, their concentration in selected profiles indicates that goals-of-care considerations frequently coexist with acute medical management.

### 4.2 Demographic and socioeconomic context

The demographic and socioeconomic findings provide additional context for these clinical profiles. The youngest age group had higher proportions of Medicaid coverage, residence in the lowest ZIP-code income quartile, documented homelessness, and Black race/ethnicity, whereas the oldest group had higher proportions of women, Medicare coverage, and residence in the highest income quartile. These differences are descriptive and were not incorporated into the clustering models; they therefore should not be interpreted as causes of the observed profiles. Future analyses should test whether payer, neighborhood income, race/ethnicity, rurality, and housing instability are independently associated with cluster membership, ED utilization, admission, mortality, or other outcomes.

### 4.3 Methodological strengths and interpretability

Methodologically, the study extends conventional comorbidity assessment by examining co-occurrence patterns rather than reducing disease burden to a single additive score. Traditional indices such as the Charlson Comorbidity Index are useful for risk adjustment but do not represent the structure of conditions that appear together (Charlson et al., 1987). Unsupervised methods can reveal such structure and generate clinically testable hypotheses (Hassaine et al., 2020, Prakash et al., 2021, Forgy, 1965). At the same time, the modest silhouette values observed for the eight-cluster solutions indicate that the profiles were not sharply separated. This is plausible in real-world multimorbidity, where diagnoses overlap across encounters, but it supports interpreting the clusters as exploratory clinical phenotypes rather than fixed or biologically discrete AD subtypes.

The heatmaps strengthened interpretability by displaying the proportion of encounters within each cluster containing each diagnosis. This made the basis for the cluster descriptions transparent and allowed high-loading diagnoses to be distinguished from less frequent contributors. Nevertheless, labels such as psychiatric-metabolic overlap or frailty with infection remain clinical summaries of administrative-code patterns, not independently validated syndromes. Replication, assessment of cluster stability across alternative feature sets and algorithms, and review by multiple clinical raters are needed before these labels can be considered robust.

### 4.4 Clinical implications

The findings have potential relevance for acute-care planning but should not yet be used as a stand-alone triage or treatment tool. Psychiatric-metabolic profiles may prompt attention to behavioral symptoms, medication use, glycemic control, renal function, and social support, whereas infection-frailty profiles may prompt assessment for delirium, dehydration, respiratory compromise, and existing goals of care (Yourman et al., 2020, Morandi et al., 2013, Pal and Manning, 2014, Pinardi et al., 2024, Kojima et al., 2018, Strehlow et al., 2024). Such applications would require prospective validation and evaluation of whether profile-informed care improves outcomes beyond standard clinical assessment.

### 4.5 Broader applicability and future directions

An additional strength is the portability of the analytic framework. The sequence of clinically meaningful stratification, binary diagnosis representation, unsupervised clustering, and heatmap-based profiling could be adapted to other large claims, discharge, or electronic health record datasets. It may also be useful for characterizing multimorbidity in other neurological, psychiatric, and chronic medical conditions and across inpatient, outpatient, and emergency settings. Adaptation would require attention to differences in coding practices, available clinical variables, sample size, and the intended clinical use.

Future research should validate these profiles in other NEDS years and independent data sources, examine their stability under different clustering parameters, and determine whether they are associated with hospital admission, mortality, revisits, resource use, or discharge disposition. Incorporating laboratory results, medications, functional status, caregiver context, and direct measures of social risk may yield more clinically informative phenotypes. Supervised classifiers or real-time decision-support tools should be considered only after the clusters demonstrate reproducibility, prognostic value, calibration, and clinical utility.

### 4.6 Limitations

This study has several limitations. NEDS is an encounter-level administrative database; diagnoses may be affected by coding error, underrecognition, and variation in documentation, and repeated visits by the same individual cannot be identified. The G30.x code identifies documented AD but does not capture disease severity, cognitive status, or functional impairment. Consequently, some age-related differences may reflect variation in AD stage rather than chronological age. The database also lacks laboratory results, medication details, caregiver availability, and direct measures of frailty or functional status.

The cluster solutions were influenced by the selected top 30 diagnoses, binary coding, the prespecified value of k = 8, and the chosen analytic parameters. Rare but clinically important conditions may have been omitted, and the modest internal separation metrics indicate substantial overlap among profiles. UMAP coordinates are visualization-dependent and should not be interpreted as clinical distances, while the heatmap labels involved clinical judgment and may vary among raters. The analyses were cross-sectional and did not establish temporal progression, causality, or associations with post-ED outcomes.

Socioeconomic variables were limited to administrative measures and area-level proxies. HCUP provides a combined race/ethnicity variable, ZIP-code income quartile does not measure individual household income, and homelessness identified through Z59.0x codes is likely underdocumented. These factors were described but not included in the clustering models. In addition, the study used a single 2022 dataset, and COVID−19-related diagnoses and exposure codes may have influenced the profiles. Finally, the unsupervised analyses were conducted on observed encounters rather than survey-weighted national estimates; therefore, cluster membership and profile frequencies should not be interpreted as nationally weighted prevalence estimates.

## 5 Conclusion

In summary, this study identified distinct, age-specific multimorbidity clusters among older adults with Alzheimer’s disease in emergency care. Using heatmap-guided interpretation of cluster loadings, we uncovered syndromic profiles spanning psychiatric, metabolic, infectious, and end-of-life domains. These patterns highlight the heterogeneous nature of AD-related ED presentations and provide a reproducible, scalable approach for clinical subtyping. The findings open new avenues for precision triage, proactive care planning, and research into age-tailored dementia interventions in acute care settings.

## Compliance with Ethical Standards

This study used de-identified, publicly available data from the Healthcare Cost and Utilization Project Nationwide Emergency Department Sample. Because the dataset does not contain individually identifiable patient information, institutional review board approval and informed consent were not required. All analyses were conducted in accordance with applicable ethical standards for research using secondary de-identified healthcare data.

## CRediT authorship contribution statement

**Tursun Alkam:** Writing – original draft, Visualization, Methodology, Formal analysis, Data curation, Conceptualization. **Ebrahim Tarshizi:** Writing – review & editing. **Andrew H Van Benschoten:** Writing – review & editing.

## Declaration of Competing Interest

The authors declare that they have no conflicts of interest relevant to this work.

## Footnotes

Appendix A Supplementary data associated with this article can be found in the online version at doi:10.1016/j.ibneur.2026.08.016.
