# Supplementary material for: Data-driven subtyping of Alzheimer’s emergency presentations using unsupervised machine learning
Source: IBRO Neurosci Rep. 2026 Aug 21;21:687–97. doi: 10.1016/j.ibneur.2026.08.016 (PMC13542608; doi:10.1016/j.ibneur.2026.08.016)
Supplement: Supplementary file 1 [file mmc1.docx]

**Figure Legend:**

**Supplementary Figure 1. Elbow Method for Determining Optimal Cluster Number (k) by Age Group.** Elbow plots display the within-cluster sum of squares (inertia) for k-values ranging from 2 to 9 across AD emergency department visits in four age groups: (A) 60-64, (B) 65-74, (C) 75-84, and (D) 85+. No distinct inflection point was observed; however, a choice of k = 8 was adopted across all groups to enhance interpretability, promote consistency, and facilitate cross-age comparisons of multimorbidity clusters.
