# Supplementary material for: Data-driven subtyping of Alzheimer’s emergency presentations using unsupervised machine learning
Source: IBRO Neurosci Rep. 2026 Aug 21;21:687–97. doi: 10.1016/j.ibneur.2026.08.016 (PMC13542608; doi:10.1016/j.ibneur.2026.08.016)
Supplement: Supplementary file 2 [file mmc2.docx]

**Supplementary Table 1. Internal cluster validation metrics across k=2–9 by age group**

Metrics computed on standardized top-30 binary ICD-10 diagnosis indicators within each age stratum. Higher silhouette and Calinski–Harabasz indicate better separation; lower Davies–Bouldin indicates better compactness/separation.

**Age group: 60–64 (n=2784)**

| **k** | **Silhouette** | **Calinski Harabasz** | **Davies Bouldin** | **Inertia** |
| --- | --- | --- | --- | --- |
| 2 | 0.1315 | 194.65 | 2.9153 | 78058.38 |
| 3 | 0.1113 | 167.56 | 3.6581 | 74537.69 |
| 4 | 0.1107 | 151.12 | 3.1937 | 71809.41 |
| 5 | 0.1061 | 137.77 | 3.1351 | 69698.79 |
| 6 | 0.0795 | 128.54 | 3.0905 | 67828.09 |
| 7 | 0.0931 | 123.26 | 2.8644 | 65954.80 |
| 8 | 0.0921 | 115.65 | 2.7474 | 64662.29 |
| 9 | 0.0729 | 110.26 | 2.8670 | 63375.51 |

**Age group: 65–74 (n=18512)**

| **k** | **Silhouette** | **Calinski Harabasz** | **Davies Bouldin** | **Inertia** |
| --- | --- | --- | --- | --- |
| 2 | 0.1627 | 1020.25 | 3.0448 | 526348.36 |
| 3 | 0.1147 | 974.32 | 2.8747 | 502460.83 |
| 4 | 0.0848 | 931.50 | 3.4870 | 482507.06 |
| 5 | 0.0817 | 875.92 | 3.3324 | 466957.80 |
| 6 | 0.0780 | 824.12 | 3.1724 | 454221.50 |
| 7 | 0.0692 | 779.93 | 3.0361 | 443265.63 |
| 8 | 0.0659 | 740.05 | 2.9563 | 433889.60 |
| 9 | 0.0654 | 714.03 | 2.8592 | 424353.37 |

**Age group: 75–84 (n=51440)**

| **k** | **Silhouette** | **Calinski Harabasz** | **Davies Bouldin** | **Inertia** |
| --- | --- | --- | --- | --- |
| 2 | 0.1624 | 3357.04 | 2.5337 | 1448655.28 |
| 3 | 0.1196 | 3084.55 | 3.5083 | 1377938.28 |
| 4 | 0.1050 | 2784.94 | 3.4076 | 1327562.68 |
| 5 | 0.0894 | 2619.96 | 3.2651 | 1281995.80 |
| 6 | 0.0822 | 2504.44 | 3.0250 | 1241053.36 |
| 7 | 0.0678 | 2393.04 | 3.0012 | 1206413.35 |
| 8 | 0.0652 | 2269.34 | 2.8986 | 1179040.49 |
| 9 | 0.0852 | 2151.38 | 2.6138 | 1156265.44 |

**Age group: 85+ (n=52717)**

| **k** | **Silhouette** | **Calinski Harabasz** | **Davies Bouldin** | **Inertia** |
| --- | --- | --- | --- | --- |
| 2 | 0.1436 | 3640.08 | 2.6673 | 1479357.46 |
| 3 | 0.1035 | 3276.68 | 3.5337 | 1406638.29 |
| 4 | 0.0928 | 2902.03 | 3.4083 | 1357333.06 |
| 5 | 0.0925 | 2692.19 | 3.1681 | 1313226.37 |
| 6 | 0.0967 | 2548.68 | 2.9649 | 1273604.78 |
| 7 | 0.0781 | 2409.30 | 2.8155 | 1241128.75 |
| 8 | 0.0764 | 2262.78 | 2.7202 | 1216072.26 |
| 9 | 0.0711 | 2153.88 | 2.7250 | 1191870.70 |

**Supplementary Table 2: Top 30 Diagnostic Codes Co-Occurred With AD Diagnosis in Age Group 60-64**

| ICD-10 codes | Description | CountS |
| --- | --- | --- |
| Z20822 | Contact with (suspected) exposure to COVID-19 | 1120 |
| I10 | Essential (primary) hypertension | 1059 |
| Z79899 | Other long-term (current) drug therapy | 829 |
| E785 | Hyperlipidemia, unspecified | 709 |
| K219 | Gastro-esophageal reflux disease without esophagitis | 457 |
| Z87891 | Long-term (current) use of opiate analgesics | 443 |
| E039 | Hypothyroidism, unspecified | 400 |
| Z7982 | Long-term (current) use of aspirin | 392 |
| N179 | Acute kidney failure, unspecified | 372 |
| Z66 | Do Not Resuscitate (DNR) status | 367 |
| E119 | Type 2 diabetes mellitus without complications | 361 |
| N390 | Urinary tract infection, site not specified | 360 |
| G40909 | Epilepsy, unsp, not intractable, without status epilepticus | 342 |
| F419 | Anxiety disorder, unspecified | 338 |
| F32A | Depression, unspecified | 327 |
| I2510 | Athscl heart disease of native coronary artery w/o ang pctrs | 312 |
| Z8673 | Personal history of drug use or rehabilitation | 307 |
| E876 | Hypokalemia | 297 |
| G9341 | Metabolic encephalopathy | 290 |
| E860 | Dehydration | 283 |
| J449 | Chronic obstructive pulmonary disease, unspecified | 264 |
| F17210 | Nicotine dependence, cigarettes, uncomplicated | 256 |
| Z794 | Long-term (current) use of insulin | 245 |
| D649 | Anemia, unspecified | 225 |
| U071 | COVID-19 | 212 |
| Z7984 | Long-term (current) use of oral hypoglycemic drugs | 212 |
| Z7901 | Long-term (current) use of anticoagulants | 203 |
| J9601 | Acute respiratory failure with hypoxia | 198 |
| A419 | Sepsis, unspecified organism | 195 |
| E1122 | Type 2 diabetes mellitus w diabetic chronic kidney disease | 183 |

**Supplementary Table 3: Top 30 Diagnostic Codes Co-Occurred With AD Diagnosis in Age Group 65-74**

| ICD-10 CODES | DESCRIPTION | COUNT |
| --- | --- | --- |
| I10 | Essential (primary) hypertension | 17378 |
| Z20822 | Contact with and (suspected) exposure to COVID-19 | 12792 |
| Z79899 | Other long-term (current) drug therapy | 11067 |
| E785 | Hyperlipidemia, unspecified | 10848 |
| Z87891 | Personal history of nicotine dependence | 8435 |
| Z7982 | Long-term (current) use of aspirin | 6470 |
| E119 | Type 2 diabetes mellitus without complications | 6387 |
| I2510 | Athscl heart disease of native coronary artery w/o ang pctrs | 5960 |
| K219 | Gastro-esophageal reflux disease without esophagitis | 5431 |
| Z7901 | Long-term (current) use of anticoagulants | 4521 |
| F17210 | Nicotine dependence, cigarettes, uncomplicated | 4219 |
| Z7984 | Long-term (current) use of oral hypoglycemics | 4156 |
| J449 | Chronic obstructive pulmonary disease, unspecified | 3840 |
| N179 | Acute kidney failure, unspecified | 3764 |
| E039 | Hypothyroidism, unspecified | 3609 |
| Z794 | Long-term (current) use of insulin | 3427 |
| E1122 | Type 2 diabetes mellitus w diabetic chronic kidney disease | 3181 |
| F419 | Anxiety disorder, unspecified | 2819 |
| U071 | COVID-19 | 2808 |
| I110 | Hypertensive heart disease with heart failure | 2785 |
| E876 | Hypokalemia | 2738 |
| N390 | Urinary tract infection, site not specified | 2603 |
| Z8673 | Personal history of transient ischemic attack (TIA) and cerebral infarction without residual deficits | 2561 |
| E7800 | Pure hypercholesterolemia, unspecified | 2535 |
| E871 | Hypo-osmolality and hyponatremia | 2421 |
| I4891 | Unspecified atrial fibrillation | 2396 |
| F32A | Depression, unspecified | 2388 |
| E669 | Obesity, unspecified | 2310 |
| I252 | Old myocardial infarction | 2169 |
| I129 | Hypertensive chronic kidney disease w stg 1-4/unsp chr kdny | 2168 |

**Supplementary Table 4: Top 30 Diagnostic Codes Co-Occurred With AD Diagnosis in Age Group 75-84**

| ICD-10 CODES | DESCRIPTION | COUNT |
| --- | --- | --- |
| I10 | Essential (primary) hypertension | 22554 |
| Z20822 | Contact with and (suspected) exposure to COVID-19 | 21821 |
| E785 | Hyperlipidemia, unspecified | 19739 |
| Z79899 | Other long-term (current) drug therapy | 15466 |
| Z66 | Do Not Resuscitate status | 11670 |
| Z87891 | Personal history of nicotine dependence | 11661 |
| I2510 | Athscl heart disease of native coronary artery w/o ang pctrs | 10096 |
| Z7982 | Long-term (current) use of aspirin | 9992 |
| K219 | Gastro-esophageal reflux disease without esophagitis | 9188 |
| N179 | Acute kidney failure, unspecified | 9169 |
| N390 | Urinary tract infection, site not specified | 8929 |
| E039 | Hypothyroidism, unspecified | 8346 |
| Z7901 | Long-term (current) use of anticoagulants | 6954 |
| G9341 | Metabolic encephalopathy | 6582 |
| E860 | Dehydration | 6521 |
| E119 | Type 2 diabetes mellitus without complications | 6493 |
| Z8673 | Personal history of transient ischemic attack (TIA) and cerebral infarction without residual deficits | 6114 |
| E876 | Hypokalemia | 6038 |
| I129 | Hypertensive chronic kidney disease w stg 1-4/unsp chr kdny | 6037 |
| F32A | Depression, unspecified | 5771 |
| E1122 | Type 2 diabetes mellitus w diabetic chronic kidney disease | 5456 |
| U071 | COVID-19 | 5093 |
| F419 | Anxiety disorder, unspecified | 4754 |
| Z7984 | Long-term (current) use of oral hypoglycemics | 4684 |
| J449 | Chronic obstructive pulmonary disease, unspecified | 4610 |
| Z515 | Encounter for palliative care | 4540 |
| J9601 | Acute respiratory failure with hypoxia | 4264 |
| I4891 | Unspecified atrial fibrillation | 4252 |
| D649 | Anemia, unspecified | 4217 |
| I110 | Hypertensive heart disease with heart failure | 4160 |

**Supplementary Table 5: Top 30 Diagnostic Codes Co-Occurred With AD Diagnosis in Age Group 85+**

| ICD-10 CODES | DESCRIPTION | COUNT |
| --- | --- | --- |
| Z20822 | Contact with and (suspected) exposure to COVID-19 | 23073 |
| I10 | Essential (primary) hypertension | 21213 |
| E785 | Hyperlipidemia, unspecified | 18685 |
| Z66 | Do not resuscitate status | 18588 |
| Z79899 | Other long-term (current) drug therapy | 15107 |
| I2510 | Athscl heart disease of native coronary artery w/o ang pctrs | 10651 |
| N179 | Acute kidney failure, unspecified | 10541 |
| E039 | Hypothyroidism, unspecified | 10294 |
| Z87891 | Personal history of nicotine dependence | 10271 |
| N390 | Urinary tract infection, site not specified | 10114 |
| Z7982 | Long-term (current) use of aspirin | 10060 |
| K219 | Gastro-esophageal reflux disease without esophagitis | 9431 |
| Z7901 | Long-term (current) use of anticoagulants | 8140 |
| E860 | Dehydration | 7474 |
| I129 | Hypertensive chronic kidney disease w stg 1-4/unsp chr kdny | 7415 |
| G9341 | Metabolic encephalopathy | 6800 |
| Z515 | Encounter for palliative care | 6516 |
| Z8673 | Personal history of transient ischemic attack (TIA) | 6511 |
| I4891 | Unspecified atrial fibrillation | 5839 |
| E876 | Hypokalemia | 5656 |
| U071 | COVID-19 | 5637 |
| I110 | Hypertensive heart disease with heart failure | 5624 |
| J9601 | Acute respiratory failure with hypoxia | 5465 |
| E119 | Type 2 diabetes mellitus without complications | 5202 |
| N1830 | Chronic kidney disease, stage 3 unspecified | 5170 |
| F32A | Depression, unspecified | 4999 |
| E1122 | Type 2 diabetes mellitus w diabetic chronic kidney disease | 4965 |
| D649 | Anemia, unspecified | 4791 |
| I480 | Paroxysmal atrial fibrillation | 4780 |
| I130 | Hyp hrt & chr kdny dis w hrt fail and stg 1-4/unsp chr kdny | 4764 |
